# Lonely places or lonely people? Investigating the relationship between loneliness and place of residence

**DOI:** 10.1186/s12889-020-08703-8

**Published:** 2020-05-27

**Authors:** Christina R. Victor, Jitka Pikhartova

**Affiliations:** 1 College of Health and Life Sciences, Department of Clinical Sciences, Kingston Lane, Brunel University London, Uxbridge, Middlesex, UB8 3PH UK; 2 Department of Epidemiology and Public Health, University College London, WC1E 6BT, London, UK

**Keywords:** Loneliness, Area, Deprivation, Urban, Rural, English Longitudinal Study of Ageing

## Abstract

**Background:**

Loneliness in later life is largely presented as a problem of the individual focusing upon antecedents such as demographic or health factors. Research examining the role of the broader living environments is much rarer. We examined the relationship between loneliness and three dimensions of the lived environment: geographical region, deprivation, and area classification (urban or rural).

**Methods:**

Our sample consisted of 4663 core members (44% males) aged 50+ (wave 7 mean age 72.8, S.D. = 7.1) present both in waves 3 (2006) and 7 (2014) of the English Longitudinal Study of Ageing (ELSA). Loneliness was measured using two approaches, individual and area-based, and both waves included these questions. Individual-based (self-reported) loneliness was assessed using the three item University of California Los Angeles (UCLA) scale (ranging from 3 = not lonely to 9 = lonely) with a score of 6+ defining loneliness. We also used a novel question which asked participants to evaluate how often they felt lonely in their area of residence (area-based; ranging from 1 = often to 7 = never, using cut off 4+ to define loneliness). The lived environment was classified in three different ways: the Index of Multiple Deprivation (IMD), Government Office Regions (GOR), and area classification (urban or rural). Covariates with established relationship with loneliness including demographic factors, social engagement and health, were included in the analyses.

**Results:**

In wave 7, the prevalence of individual-based loneliness was 18% and area-based was 25%. There was limited congruence between measures: 68% participants reported no individual- or area-based loneliness and 9% reported loneliness for both measures. After adjusting for individual co-variates only one significant relationship was observed between loneliness and area -based characteristics. A significant association was observed between area-based loneliness and deprivation score, with higher levels of loneliness in more deprived areas (OR = 1.4 for highest quintile of deprivation).

**Conclusions:**

Our results indicate that loneliness in older adults is higher in the most deprived areas independent of individual-level factors. In order to develop appropriate interventions further research is required to investigate how area-level factors combine with individual-level loneliness vulnerability measures to generate increased levels of loneliness in deprived areas.

## Background

### Loneliness

Loneliness as a concept is characterised as the gap between the aspirations and reality of an individual’s quality, quantity, and /or mode of social relationships, or some combination of these elements which is ‘unwanted’ by the individual. These criteria serve to differentiate loneliness from other concepts, most notably social isolation and living alone [1, 2]. Contemporary evidence from the UK reports that the highest prevalence of loneliness, defined as those reporting being often/always lonely, is observed in young adults aged 16 to 24 (10%) and that there is an age-related decline with 3% of those aged 75+ reporting that they are ‘often/always’ lonely [3–5].

Loneliness is considered a public health issue because of the association with a range of negative outcomes including decreased well-being and quality of life [6], increased risk of deteriorating physical [7, 8] and mental health [9, 10], and increased mortality [11, 12] alongside unhealthy behaviours [13–15] and health and social care services utilisation [16]. Loneliness has attracted the attention of policy makers and service providers leading to extensive investment in loneliness interventions although evidence of effectiveness remains limited [17–20].

### Factors related to loneliness in later life

Victor and Sullivan [21] have argued that loneliness in later life is the product of the interaction of three sets of factors operating at macro- (societal), meso- (community/neighbourhood) and micro- (individual) level. Our current understanding of the antecedents of loneliness in later life emphasises micro-level factors, such as psychological (e.g. self-esteem), life-events (e.g. bereavement) or the onset of chronic illness, thereby positioning loneliness as a problem of the individual. Victor and Sullivan [21] suggest including the broader meso- and macro-level characteristics of older people lives, and their intersection with the micro-level factors, to generate a comprehensive understanding of the genesis of loneliness. Emblematic of the evidence gap, regarding the importance of meso- and macro- level elements in understanding loneliness in later life, is our limited understanding of the geography of loneliness and how area level factors relate to loneliness.

At the macro-level, there is comparatively little research examining the distribution of loneliness geographically and considering how area based factors, such as deprivation or rural/urban typology are associated with loneliness in later life [22]. Age UK, the UK’s largest charity for older people, produced loneliness ‘heat maps’ for 32,844 neighbourhoods in England [23] to facilitate targeting resources at areas most ‘in need’. Dorling [24] produced ‘loneliness maps’ for Great Britain. Both studies did not use loneliness per-se but rather ‘relative risk of loneliness’ using factors associated with loneliness such as age, household size, marital status, and self-reported health status. Neither study considered how and why loneliness may vary between different types of geographical areas.

At the meso- level, Nyquist and colleagues demonstrated in the Finnish context that older individual’s external meso-level context, such as neighbourhood quality and trust, was associated with loneliness [22]. They posit that neighbourhood and community are important to older people as ‘social resource’ and act as bulwark against loneliness because they are not linked into employment-based networks and social resources. Similarly Kearns and colleagues, using data from adult householders in Glasgow, U.K., and Domenech-Abella and colleagues, using population based sample from Barcelona, Spain, report that loneliness is lower where residents rated their neighbourhood environment, social capital and individual social capital higher [25, 26]. None of these studies considered how the specific types of geographical area such as urban, rural or deprived were linked with loneliness.

### The geography of loneliness

We identified three types of studies that focus upon loneliness and area type: descriptions of loneliness in specific areas e.g. deprived or rural areas; comparisons of loneliness between different area types (e.g. by levels of deprivation or between urban/rural areas), and investigations of area types as a risk factor for loneliness.

Descriptive studies of older adults in the UK report high levels of loneliness in deprived areas. A study of 65 participants, mean age 71.6 (range 51–92), living in deprived areas of Manchester reported the prevalence of loneliness as 16% (severe) and 49% (moderate) using the 11 items de Jong Gierveld scale [27]. Using the same measure, Scharf and de Jong Gierveld reported a prevalence of loneliness of 13% (severe) and 43% (moderate) for 497 participants aged 60–96 (mean age = 71.6) living in 3 urban disadvantaged areas in Britain (London, Liverpool and Manchester) [28]. De Koning et al. reported a prevalence of 9% for loneliness for those aged 65+ in 6 rural areas of England [29]. This approximates to the national norm [﻿5﻿] and is comparable with findings reported by Wenger and Burholt (2004) in a rural area of North Wales [30].

Studies drawing comparisons of individual-based loneliness between deprived and non-deprived areas or rural and urban areas for older adults are less numerous. At a national level, Menec and colleagues evaluated the distribution of loneliness across Canada, using a single item from the CES-D asking about loneliness in the last week and reported similar loneliness prevalence in rural (9%) and urban (10%) areas [﻿﻿31﻿]. Havens and Hall (2004) compared loneliness among older (aged 72+) Manitobans using a customised measure (derived from the de Jong Gierveld scale and two single-item questions used elsewhere), and reported loneliness prevalence of 47% (rural) and 43% (urban) [32]. A Polish study, reported by Tobiasz-Adamczyk, of 1200 older adults aged 65+ concluded that there were no differences in loneliness between urban and rural areas using the 3 item UCLA scale (14.7% (urban) versus 15.8% (rural)) [33].

Evidence for deprivation or area typology (urban/ rural) as a risk factor for loneliness in older adults is sparse. Cross-sectional data from Ireland reports crude mean UCLA loneliness scores that are the same for urban and rural areas (mean of 4.1, range 3–9) but posit that rurality played a role in the complex pathway to loneliness for older adults [34﻿]. Given the limited and inconsistent empirical evidence we sought to examine the relationship between loneliness and three distinct types of geographical area: deprivation, geographical region, and area typology (urban or rural) among older people living in England.

## Methods

### Dataset

We address our research question using the English Longitudinal Study of Ageing (ELSA): a nationally representative survey of approximately 10,000 people aged 50+ years living in England, modelled on the well-established US Health and Retirement Survey (HRS; http://hrsonline.isr.umich.edu). The wave 0 ELSA cohort consisted of individuals who participated in the 1998, 1999, and 2001 waves of Health Survey for England (HSE; https://www.ucl.ac.uk/hssrg/studies/hse). Those who were aged 50+ in 2002 were invited to take part in the ELSA baseline (wave 1) which included an administered survey and a self-completion questionnaire. Subsequently data collection for ELSA has taken place at two-yearly intervals with the most recently available data for analysis being from wave 8 (2016). To compensate for attrition the ELSA sample has been refreshed with HSE participants aged 50 years in waves 3, 4, 5 and 6. Ethical approval was granted from the National Research and Ethics Committee. Further details about the design, sampling and methodology of ELSA are available elsewhere (http://www.elsa-project.ac.uk/documentation).

### Measures

#### Loneliness

We included two measures of loneliness in our analysis with dichotomisation to classify those who are lonely defined using the upper quartile of the distributions. Our main outcome variable is the revised UCLA loneliness scale [35] consisting of three items: “How often do you feel you lack companionship?” “How often do you feel isolated from others?”, and “How often do you feel left out?” Participants selected their response from three options (hardly ever/never; some of the time; often) which are coded 1–2-3 and summed to give a total score ranging from 3 and 9. Scores are dichotomised, with scores 3–5 classified as not lonely and 6+ as lonely. Waves 3 and 7 of ELSA included a suite of questions focused on subjective neighbourhood evaluation [36]. This included a question which asked participants to agree or disagree with the statement ‘I often feel lonely living in this area’ using a 7-point Likert scale. This single item has not, to our knowledge, been used elsewhere to evaluate feelings of loneliness within an explicit spatial context. The scale was recoded for consistency of directionality with the UCLA measure and dichotomised with a score of 4+ defining loneliness. As the use of this measure of loneliness has not has not previously been reported we undertook the evaluation of the measure in terms of its relationship with established loneliness predictors to determine its utility. This analysis was undertaken for both waves 3 and 7 to test stability of the relationships. Both measures were included in the self-completion questionnaire component of data collection and thus there are issues of missing data.

ELSA data routinely includes details of the 9 administrative regions of England. No more fine-grained data about the areas in which participants live are routinely provided to assure participants’ anonymity and confidentiality. Following the approval of a special data access request the National Centre for Social Research (NatCen) (http://natcen.ac.uk/), the organisation that oversees such matters, provided us with a data set which included two area based measures (urban/rural classification; deprivation index) which were linked to individuals via their individual study number for wave 6 of ELSA.

The urban and rural area classification is produced by the Office for National Statistics (ONS) and areas are defined as Urban (includes urban areas, towns and urban fringes) and Rural (village, isolated dwellings/hamlets) based upon the size of the settlement and population density (https://www.gov.uk/government/collections/rural-urban-classification). Our provided data set were grouped into 4 categories: urban, town and fringe, village, and hamlets and isolated dwellings. The Index of Multiple Deprivation (IMD) has been produced and validated by the Department of Communities and Local Government. It is area-level measure available for 32,844 small areas in England based upon 7 domains of deprivation (Income; employment; education, skills and training; health deprivation and disability; crime; barriers to housing and services; and living environment) (https://data.gov.uk/dataset/imd_2004). Areas are ranked by score. As there is no absolute value that differentiates deprived from non-deprived areas analysis is usually undertaken using quintiles with 1st quintile characterising the least deprived area and 5th quintile the most deprived.

#### Covariates

The covariates included in our analysis are well-established predictors of loneliness: gender (coded as males/females), marital/partnership status (married/in partnership, always single, divorced/separated, widowed/partner died), social relationships (close relationships with 2+ family/friends and current participation in civic activities), employment status (out of job market, employed/self-employed, looking after home/family), health (self-rated health (excellent/very good/good, fair/poor; used from wave next to baseline, as in baseline it was not collected), depressive symptoms (CESD-8 scale dichotomised as 0-2 positive responses vs 3+ out of 8 questions), activities of daily living (ADL/IADL dichotomised as no difficulties with activities of daily living vs. at least one difficulty) and long-standing limiting illness (no, yes but not limiting, yes and limiting). We excluded co-variates such as income that were included in the IMD measure.

### Analytic sample

Our analysis is based on 4663 ELSA core members that satisfied two criteria: (a) they participated in waves 3 and 7 (collected in 2006 and 2014; which both included the UCLA scale and also the area-based loneliness measure) and (b) they reported the same address in wave 7 as in wave 6 (as area characteristics data was only available for wave 6). Only 5% of those who participated in waves 3 and 7 changed address between waves 6 and 7, therefore our analytical sample represents 95% of those who took part in both waves 3 and 7.

### Analysis plan

Our analysis plan consisted of two phases. First, we used descriptive statistics and bivariate regression analysis to profile our analytic sample and to evaluate the crude association between independent (loneliness) and dependent variables separately for both wave 3 and wave 7 and to evaluate the utility of the area-based loneliness measure. We then undertook a series of multivariable regression analyses to evaluate the role of area characteristics in reported loneliness. We used the complete case sample as we considered it inappropriate to use imputation for missing socio-demographic data. Our three regression models were as follows: model A, loneliness measured by the 3-item UCLA scale adjusted for age and gender; model B, further adjusted for social network characteristics (marital status, evaluation of close feelings to at least two members of the family or friends, civic participation, job market participation, UCLA-measured loneliness in baseline (wave 3), and mutually adjusted for area loneliness (when analysing individual loneliness, we have adjusted also for area-based loneliness and vice versa)); model C, further adjusted for health status characteristics (depressive symptoms, long-standing limiting illness, self-rated health, and difficulties with activities of daily living). We repeated this analysis using the area-based loneliness measure as our outcome. Analyses were carried using STATA MP Version 13.0 with *p*-value < 0.05 signifying statistical significance.

## Results

### Characteristics of sample

Table 1 describes the characteristics of our analytical sample. The mean age of participants in wave 7 was 72.8 years (S.D. =7.1), 56% were females and 22% participants were widowed. The majority, 84%, were retired, had two and more close relationships with family/other relatives/friends/neighbours or colleagues (88%), and were socially active (74%). In terms of health status, 70% rated their health as good, 20% reported depressive symptoms, and 40% stated that they had a longstanding (12 months or longer) illness that limited daily activities. England is a predominately urban society and most (72.6%) ELSA participants lived in areas classified as urban:11.2% lived in most deprived areas and 26% in lowest deprivation quintile (for cross-tabulation see Additional file 1: Table S7). In terms of government administrative regions, 17% were resident in the South East and all other regions had around 10% of ELSA participants. Key differences in our analytic sample characteristics between waves 3 and 7 reflect the effects of temporal changes in the population including the increased mean age of the wave 7 participants (72.8 compared to 65.5); higher levels of widowhood (22% compared with 15%); increased number of retired (84% vs 58%) and higher levels of reported longstanding limiting illness (40% vs 31%) (see Additional file 1: Table S1 for a comparison of waves 3 and 7).

**Table 1:** Descriptive table of analytical sample

*N* = 4663		N (%)/mean (S.D)	Individually-based loneliness (%)	Area-based loneliness (%)
Gender	Males	43.8%	13.7	19.3
	Females	56.2%	20.6	28.8
Age	**Wave 3**	65.5 (7.9)	19.3	24.8
	**Wave 7**	72.8 (7.1)	17.6	24.6
**SOCIAL NETWORK**				
Marital status	(Re) married/partnership	2903 (62.3)	10.9	19.5
	Always single	207 (4.4)	23.7	24.7
	Divorced/separated	510 (10.9)	28.5	33.7
	Widowed/partner died	1043 (22.4)	31.5	36.1
	*Missing (N)*	*0*		
Has close relationship with 2+ family member/friend	Yes	3528 (87.5)	15.4	23.2
	No	506 (12.5)	33.8	35.1
	*Missing (N)*	*629*		
Active civic participation	Yes	2825 (74.2)	15.4	21.8
	No	983 (25,8)	22.6	30.0
	*Missing*	*855*		
Part of job market	Out of job market	3928 (84.4)	18.1	25.0
	Employed/semi-employed	530 (11.4)	13.2	20.2
	Looking after home/family	194 (4.2)	19.5	29.3
	*Missing*	*11*		
**HEALTH STATUS**				
Self-rated health (wave 4)	Excellent /very good/good	3125 (70.3)	13.3	21.3
	Fair/poor	1318 (29.7)	29.1	33.5
	*Missing*	*220*		
Depressive symptoms	No	3524 (80.4)	10.9	20.4
	3+ out of 8	860 (19.6)	47.7	42.7
	*Missing*	*279*		
Activities of daily living (ADL/IADL)	No difficulties	3433 (73.6)	14.4	22.4
	Difficulty with 1+	1228 (26.4)	28.6	32.0
	*Missing*	*2*		
Long-standing illness	No	1848(39.7)	13.4	20.4
	Yes, not limiting	947 (20.3)	14.8	24.0
	Yes, limiting	1866 (40.0)	23.8	29.6
	*Missing*	*2*		
Reported loneliness (UCLA scale)	No	3208 (82.5)	n/a	18.1
	Yes (6+ out of 9)	683 (17.6)		54.0
	*Missing*	*772*		
Feel lonely living in this area	No	2993 (75.4)	10.6	n/a
	Yes (4+ out of 7)	957 (24.6)	38.6	
	*Missing*	*773*		
**GEOGRAPHICAL CHARACTERISTICS (wave 6)**				
Index of Multiple Deprivation (IMD)	1st quintile (least deprived)	1163 (26.0)	13.3	19.4
	2nd	1181 (26.4)	17.7	23.8
	3rd	899 (20.1)	17.4	24.3
	4th	735 (16.4)	19.5	28.9
	5th	500 (11.2)	23.2	31.6
	*Missing*	*185*		
Urban/rural distribution	Urban	3271 (72.6)	17.7	25.3
	Town/fringe	565 (12.5)	17.7	21.0
	Village	497 (11.0)	15.1	23.1
	Hamlets /isolated dwellings	174 (3.9)	15.8	23.3
	*Missing*	*156*		
Geographical regions	London	385 (8.6)	13.0	25.9
	North East	276 (6.1)	14.6	20.1
	North West	503 (11.2)	18.3	21.8
	Yorkshire and The Humber	487 (10.8)	19.5	26.4
	East Midlands	478 (10.6)	18.8	22.0
	West Midlands	494 (11.0)	19.6	23.4
	East of England	580 (12.9)	15.6	26.5
	South East	761 (16.9)	15.5	25.5
	South West	532 (11.8)	20.1	25.4
	*Missing*	*167*		

To put our analytic sample into context so that we can draw robust generalisation from our findings we compared it with total wave 7 data (not presented in the table). Our analytic sample is older (mean age of 72.8 vs 65.4), has significantly more widows (22% v 15%), the retired (84% v 63%), and has higher levels of longstanding illness. These differences reflect the impact of the ELSA sample refreshment strategy where new participants aged 50 were added (in waves 3,4,5, and 6) [36] and are reported elsewhere [37].

Comparison with those who did not take part in our waves of interest (dropped out or were added as a sample boost in later waves and therefore were not eligible for our analysis) shows that they were significantly younger, less likely to be widowed, had better health (self-rated, difficulties with activities of daily living and long-standing limiting illness) and greater access to people who could provide help/advice. No differences between the two groups (those who remain in the study and those who dropped-out between wave 3 and wave 7) were observed for presence of depressive symptoms or both individual-based and area-based loneliness (see Additional file 1: Table S2).

### Loneliness and area: bivariate analysis

The prevalence of individual and area based loneliness were stable across both waves of data collection. In wave 7, 18% were categorised as lonely people (a score of six or more on individual-based loneliness scale (the UCLA scale; lonely people) and 25% reported that they experienced area-based loneliness (lonely places) compared with 19 and 25% respectively for wave 3. The degree of agreement between our two measures of loneliness was also similar in both waves of data collection. In wave 7, 67.7% of respondents were classified as not lonely (when measured by UCLA scale) and did not report area-based loneliness and 67% in wave 3. Alternatively 9.4 and 10.7% of respondents were classified as lonely on both measures in waves 7 and 3, respectively (Additional file 1: Table S6).

Crude loneliness prevalence rates suggest an increase with deprivation for individual loneliness from 13% (least deprived) to 23% (most deprived) and 19 and 32% respectively for area loneliness (Table 1). No clear relationship with urban rural classification or region is evident. Our bi-variate regression analysis supports this initial finding. Those living in the areas characterised by the highest level of deprivation are approximately twice as likely to report experiencing both type of loneliness compared with those residents in the least deprived areas (Additional file 1: Table S4). No significant relations were observed with urban/rural characteristic of area, and only limited association with geographical regions (in terms of UCLA loneliness scale) (Additional file 1: Tables S4, S5).

Using bivariate regression analyses there was consistent relationship with gender, marital status, social relationships and health for both measures and time points (Additional file 1: Table S3). These results are reflective of accepted relationships between individual factors and loneliness and demonstrate the utility of our area based measure. We tested for gender as potential effect modifier but found no statistically significant evidence for either individual loneliness (*p*-value = 0.431), nor area-based loneliness (*p*-value = 0.439). Hence we present our results adjusted for gender, rather than stratified.

### Loneliness and area: multivariable analysis

To test if the relationships between loneliness and deprivation observed in our bivariate analysis was simply an artefact of the socio-demographic profile of these areas we undertook multivariable analysis. Three multivariable models (A, B, and C) are presented in Tables 2 and 3. For individual loneliness, model A suggests a relationship between loneliness and deprivation but this loses significance once social engagement (model B) and health status I (model C) are included. The area-based loneliness measures relationship with deprivation is attenuated but remained significant in the fully adjusted model (C). Participants living in the most deprived areas (bottom quintile) were by 53% more likely to report experiencing area-based loneliness compared with their counterparts in the least deprived areas independent of differences in socio-demographic profile, social engagement and health status (OR = 1.53, 95% CI = 1.09–2.14, *p*-value = 0.015). The model without mutual adjustment for individual-based loneliness (not presented in table) showed very similar results (OR = 1.55, 95%CI = 1.16–2.15, *p*-value = 0.009) suggesting that area characteristics have an independent influence on area-based loneliness after considering individual-based demographic, social and health characteristics.

**Table 2:** Association between geographical characteristics and *individual-based loneliness* (multilevel logistic regression)

		Model A	Model B	Model C
		OR (95%CI), *p*-value	OR (95%CI), *p*-value	OR (95%CI), *p*-value
Index of Multiple Deprivation (IMD) (ref = 1st quintile –least deprived)	2nd quintile	1.44 (1.13–1.85), 0.004	1.26 (0.97–1.64), 0.087	1.17 (0.88–1.56), 0.281
	3rd quintile	1.36 (1.05–1.78), 0.021	1.28 (0.97–1.70), 0.086	1.30 (0.97–1.76), 0.083
	4th quintile	1.54 (1.17–2.04), 0.002	1.19 (0.88–1.60), 0.250	1.09 (0.78–1.50), 0.607
	5th quintile-most deprived	2.0 (1.48–2.71), < 0.001	1.38 (0.99–1.92), 0.056	1.13 (0.78–1.61), 0.520
	*p-value for trend*	*< 0.001*	*0.096*	*0.532*
Urban/rural character (ref = Urban)	Town and fringe	0.99 (0.76–1.28), 0.923	1.10 (0.83–1.45), 0.503	1.13 (0.84–1.51), 0.433
	Village	0.84 (0.63–1.12), 0.240	0.97 (0.71–1.32), 0.836	1.04 (0.74–1.44), 0.828
	Hamlet and isolated dwellings	0.91 (0.59–1.42), 0.688	1.18 (0.74–1.88), 0.472	1.44 (0.88–2.34), 0.147
	*p-value for trend*	*0.300*	*0.626*	*0.220*
Geographical regions (ref = London)	North East	1.23 (0.74–2.06), 0.422	1.77 (0.94–3.30), 0.078	1.65 (0.85–3.19), 0.138
	North West	1.43 (0.92–2.23), 0.111	1.67 (0.94–2.95), 0.081	1.54 (0.86–2.79), 0.149
	Yorkshire and The Humber	1.68 (1.09–2.61), 0.019	2.02 (0.94–2.95), 0.013	1.97 (1.10–3.51), 0.21
	East Midlands	1.53 (0.98–2.39), 0.019	1.83 (1.03–3.25), 0.038	1.73 (0.95–3.13), 0.068
	West Midlands	1.60 (1.02–2.50), 0.038	2.19 (1.24–3.86), 0.007	2.12 (1.19–3.80), 0.011
	East of England	1.27 (0.83–1.95), 0.271	1.57 (0.90–2.74), 0.112	1.53 (0.87–2.72), 0.110
	South East	1.27 (0.83–1.98), 0.271	1.52 (0.89–2.62), 0.123	1.48 (0.85–2.56), 0.211
	South West	1.74 (1.12–2.70), 0.012	2.35 (1.35–2.94.083), 0.002	2.51 (1.43–4.43), 0.001

Model A = geographical characteristics+age + gender

Model B = Model A+ social network + individual-based loneliness from baseline wave

Model C = Model B + health characteristics

**Table 3:** Association between geographical characteristics and *area-based loneliness* (multilevel logistic regression)

		Model A	Model B	Model C
		OR (95%CI), *p*-value	OR (95%CI), *p*-value	OR (95%CI), *p*-value
Index of Multiple Deprivation (IMD) (ref = 1st quintile-least deprived)	2nd quintile	1.34 (1.08–1.67), 0.008	1.14 (0.88–1.47), 0.321	1.11 (0.86–1.44), 0.406
	3rd quintile	1.31 (1.03–1.65), 0.025	1.27 (0.97–1.66), 0.087	1.27 (0.97–1.67), 0.081
	4th quintile	1.61 (1.26–2.06), < 0.001	1.27 (0.95–1.1770), 0.106	1.26 (0.94–1.69), 0.123
	5th quintile-most deprived	1.95 (1.49–2.57), < 0.001	1.57 (1.12–2.20), 0.008	1.53 (1.09–2.14), 0.015
	*p-value for trend*	*< 0.001*	*0.006*	*0.009*
Urban/rural character (ref = Urban)	Town and fringe	0.79 (0.62–1.01), 0.065	0.78 (0.59–1.04), 0.096	0.77 (0.57–1.03), 0.085
	Village	0.98 (0.77–1.25), 0.880	1.20 (0.90–1.60), 0.195	1.26 (0.95–1.67), 0.105
	Hamlet and isolated dwellings	0.92 (0.63–1.35), 0.673	0.96 (0.61–1.50), 0.874	1.00 (0.63–1.60), 0.984
	*p-value for trend*	*0.427*	*0.790*	*0.506*
Geographical regions (ref = London)	North East	0.74 (0.48–1.12), 0.157	0.76 (0.44–1.25), 0.291	0.76 (0.45–1.26), 0.291
	North West	0.73 (0.51–1.06), 0.094	0.66 (0.42–1.02), 0.062	0.66 (0.42–1.02), 0.062
	Yorkshire and The Humber	0.98 (0.69–1.40), 0.908	0.88 (0.58–1.36), 0.576	0.89 (0.60–1.36), 0.576
	East Midlands	0.78 (0.54–1.13), 0.196	0.70 (0.45–1.09), 0.116	0.70 (0.45–1.09), 0.116
	West Midlands	0.82 (0.54–1.13), 0.287	0.71 (0.45–1.10), 0.127	0.71 (0.52–1.13), 0.190
	East of England	1.02 (0.73–1.42), 0.881	0.97 (0.64–1.46), 0.878	1.09 (0.45–1.10), 0.127
	South East	0.96 (0.68–1.29), 0.761	0.91 (0.61–1.33), 0.619	0.97 (0.63–1.46), 0.879
	South West	0.95 (0.67–1.34), 0.761	0.83 (0.54–1.27), 0.398	0.91 (0.61–1.33), 0.861

Model A = geographical characteristics+age + gender

Model B = Model A+ social network + individual-based loneliness from baseline wave

Model C = Model B + health characteristics

## Discussion

Research examining the antecedents of loneliness in older adults have predominantly focused upon individual characteristics. In our study we moved the focus away from individuals to the types of area in which they live as community/meso-level factors are neglected in loneliness research. We aimed to add to the existing evidence base by focusing upon the importance of place and the environment in which people live as potential loneliness vulnerability factors. We investigated the importance of three geographical categories in relation to loneliness: area typology (urban/rural), geographical region, and deprivation. We used two measures of loneliness: the 3-item UCLA scale (measuring self-reported personal loneliness status), and a measure focused upon ‘loneliness based on the area of residence’. We show that there are no relationships with region or area type or deprivation for the UCLA scale. However there was, after adjustment for confounding factors, a statistically significant relationship between area-based loneliness and deprivation.

Existing research focused upon understanding the prevalence of, and risk factors for, loneliness has largely concentrated on seeking explanation at the individual level. Sullivan et al. (2016) have discussed many of the limitation of this approach to studying loneliness including the presumption of shared understanding and the dynamic nature of the experience combined with the complexity and difficulty people may have in describing the experience of loneliness [﻿38﻿]. There is also an increasing acceptance that loneliness is not a static experience but one which may fluctuate during a day, a week or a year [39] and that the population characterised as ‘lonely’ is not homogeneous but includes those for whom loneliness is an enduring part of their life whilst for others loneliness may increase or decrease as they age.

We used two waves (3 and 7) of the English Longitudinal Study of Ageing (ELSA) to consider the relationship between individual loneliness and area-based loneliness as this latter question was included in these waves of data collection. In terms of our two loneliness outcome measures the revised UCLA scale is well established. The question on evaluating loneliness in the area where participants live was not and has not, to our knowledge, been used elsewhere. However, it does offer a novel insight into the experience of loneliness by locating it in the area in which people live.

Our three measures of area characteristics are all designed for administrative rather than research purposes. The measure of deprivation is designed for use as a means of targeting resources to areas in need. The urban-rural classification is limited in that it does not distinguish large conurbations such as London from smaller urban areas. Furthermore, our data show that England is a predominantly urban society and thus we may have had too few participants from rural areas making our study insufficiently powered to identify any differences. The third area characteristics was the geographical regions classification which gives only broad information from which part of England the participant comes but could help with distinguishing London area from the others regions. These caveats frame the confidence that we can have in our overall findings and highlight some issues to be addressed in further research.

Levels of loneliness, as assessed by our two measures, were broadly stable at 18% for the individual-based loneliness and 25% for the area-based one in both waves (Additional file 1: Table S1). Inviting participants to evaluate loneliness in the context of the area where they lived generated a higher level of loneliness and this finding is, to our knowledge, novel in the literature. The congruence between the measures was good for those reporting that they were not lonely but was under 50% of those reporting loneliness (Additional file 1: Table S6 a, b). This suggests that the area-based measure is extending into a domain of loneliness not embraced by the social/emotional relationship focus of the items included in the UCLA scale. Clearly the potential of the area-based measure and characteristics of those who report individual-based loneliness in one but not both measures merits further investigation.

Drawing comparisons with previous research is complex because of differences in how area typologies are defined, and loneliness is measured. Once other factors were considered, loneliness was not associated with region or area classification in terms of urban/rural. This lack of an association with urban/rural area classification aligns with the studies from Ireland [22], Canada [32] and Poland [32]. Our study reported increased prevalence of loneliness in deprived areas which, whilst not reaching the levels reported by Scharf and de Jong Gierveld [28] of 57%, are significantly higher than those in the least deprived areas (range 36–80% depending on measure and data collection wave). Once socio-demographic, social and health characteristics were considered the significant relationship between area-based loneliness and high levels of deprivation remained robust. This may reflect the features of the specific environment such as terrain or amenities, demographic characteristics, housing conditions, high crime rates, potential opportunities for engagement or issues of trust and neighbourliness and population turn-over. However as with the related concept of resilience there is a need to embrace the role of macro- (societal) and meso- (community/neighbourhood) factors in the emergence of vulnerability to loneliness. Further research with older people living in these types of areas is required to understand what is driving this relationship and what interventions might ameliorate it and to understand how micro-, meso-, and macro- level factors combine to protect or render older people vulnerable to loneliness.

### The strength and limitations

The strength of our study is rooted in two key areas: our research questions and the use of the ELSA data set. ELSA is currently the largest, most representative and longest established longitudinal study of older people in the community within the UK and, as such, is the best UK-based data set. The development of longitudinal studies of ageing, using the same model as ELSA, in Northern Ireland (The Northern Ireland Longitudinal Study of Ageing-NICOLA study established in 2012) and Scotland (Healthy Ageing in Scotland-HAGIS study completed a pilot study in 2017) include more rural areas than England and thus offer the potential to develop our analysis in the future. Our research questions attempt to extend our understanding of loneliness beyond the individual and extend it to the area in which they live. To the best of our knowledge, the question on area-based loneliness has not been used in other research and, as such, adds to our understanding of the complexity of the experience of loneliness. By including both wave 3 and wave 7 in our analysis we have been able to establish the utility of the measure as a complementary method to the individual measure. In addition, the construction of our analytic cohort enables us to include previous loneliness experiences from wave 3 in our predictive models based on wave 7. This is important as much loneliness modelling does not take into account past experiences.

Nevertheless, there are limitations to our study which relate to the conduct of ELSA including attrition, missing data because the loneliness questions are included in the self-completion rather direct interview element of ELSA and the exclusion of those older people living in care homes. More specifically the area-based loneliness question did not offer guidance to respondents in terms of the size of the area to which the question refers. The interpretation of results regarding deprivation are preliminary given that the data provided relate to 2004 and our data collection to 2006 (wave 3) and 2014 (wave 7) and there might have been changes in deprivation profile for some areas. Given the positive relationship between loneliness and deprivation it would have been useful to conduct a longitudinal analysis of how changes in deprivation linked to changes in loneliness at individual and area level. We were unable to obtain details of area classification for both time points thereby precluding a cohort study. However, this is a potential area for future research as is qualitative a finer grained quantitative research to see which elements of the deprivation measure are important in explaining the link with loneliness.

## Conclusions

Our study offers new insights into our understanding of the experience of loneliness in later life both substantively and conceptually. Empirically we show that for those aged 50 years and older the area-based measure of loneliness was independently associated with deprivation but not the urban/rural classification. This suggests that the quality of the locality in which people live, as measured by deprivation, has an independent effect on loneliness. Current interventions for loneliness are focused upon individuals and show little evidence of effectiveness [20]. Reductions in deprivation may, in theory at least, offer the potential to reduce levels of loneliness. Conceptually our study suggests that the area-based measure of loneliness is tapping into a domain of individual-based loneliness that is not covered by the social/emotional/existential domains included in key loneliness measures.

## Supplementary information

**Additional file 1: Table S1.** Comparison of ELSA sample in waves 3 and 7. **Table S2.** Comparison of wave 7 of analytical sample (those participating in waves 3, 6, and 7) and those who dropped-out. **Table S3.** Bivariate regression analyses between two measures of loneliness and socio-demographic characteristics. **Table S4.** Bivariate regression analyses between two measures of loneliness and geographical characteristics. **Table S5.** Loneliness by geographical characteristics in waves 3 and 7. **Table S6a**, **b**. Cross-tabulation of reported loneliness measures (R-UCLA and ‘Often feel lonely living in this area’) by wave (N (%). **Table S7.** Cross-tabulation of urban/rural characteristic and IMD (in %), wave 6.

## Data Availability

The ELSA data that support the findings of this study are available from UK Data Archive and National Centre for Social Research (NatCen). Restrictions apply to the deprivation and area classification data which were provided under license for the current study, and so are not publicly available.

## Abbreviations

ELSA: English Longitudinal Study of Ageing
UCLA: University of California Los Angeles
HRS: Health and Retirement Survey
HSE: Health Survey for England
NatCen: National Centre for Social Research
IMD: Index of Multiple Deprivation
