# Supplementary material for: Lonely places or lonely people? Investigating the relationship between loneliness and place of residence
Source: BMC Public Health. 2020 May 27;20:778. doi: 10.1186/s12889-020-08703-8 (PMC7251825; doi:10.1186/s12889-020-08703-8)
Supplement: Supplementary file 1 — Additional file 1: Table S1. Comparison of ELSA sample in waves 3 and 7. Table S2. Comparison of wave 7 of analytical sample (those participating in waves 3, 6, and 7) and those who dropped-out. Table S3. Bivariate regression analyses between two measures of loneliness and socio-demographic characteristics. Table S4. Bivariate regression analyses between two measures of loneliness and geographical characteristics. Table S5. Loneliness by geographical characteristics in waves 3 and 7. Table S6a, b. Cross-tabulation of reported loneliness measures (R-UCLA and ‘Often feel lonely living in this area’) by wave (N (%). Table S7. Cross-tabulation of urban/rural characteristic and IMD (in %), wave 6. [file 12889_2020_8703_MOESM1_ESM.docx]

**Supplementary file, table 1: Comparison of ELSA sample in waves 3 and 7**

|  |  | Wave 3 | Wave 7 |
| --- | --- | --- | --- |
|  |  | N (%) | N (%) |
| Gender |  |  |  |
|  | Females | 56.5% | 56.2% |
|  |  |  |  |
| Age | Mean (S.D.) | 65.5 (7.9) | 72.8 (7.1) |
| **SOCIAL NETWORK** | | | |
| Marital status | N | 4306 | 4663 |
|  | (Re)married/partnership | 2970 (69.0) | 2903 (62.3) |
|  | Always single | 209 (4.9) | 207 (4.4) |
|  | Divorced/separated | 472 (11.0) | 510 (10.9) |
|  | Widowed/partner died | 655 (15.2) | 1,043 (22.4) |
|  | *Missing (N)* | *357* | *0* |
| Has close relationship with 2+ family member/friend | N | 3907 | 4034 |
|  | Yes | 3747 (95.9) | 3528 (87.5) |
|  | *Missing (N)* | *756* | *629* |
| Active civic participation | N | 3723 | 3808 |
|  | Yes | 2835 (76.2) | 2825 (74.2) |
|  | *Missing(N)* | *940* | *855* |
| Part of job market | N | 4301 | 4652 |
|  | Out of job market | 2511 (58.4) | 3928 (84.4) |
|  | Employed/semi-employed | 1428 (33.2) | 530 (11.4) |
|  | Looking after home/family | 362 (8.4) | 194 (4.2) |
|  | *Missing(N)* | *362* | *11* |
| **HEALTH CHARACTERISTICS** | | | |
| Self-rated Health | N | n/a | 4443 |
|  | Excel/v good/good |  | 3125 (70.3) |
|  | Fair/poor |  | 1318 (29.7) |
|  | *Missing(N)* |  | *220* |
| Depressive symptoms | N | 4251 | 4384 |
|  | Yes (3+ out of 8) | 787 (18.5) | 860 (19.6) |
|  | *Missing(N)* | *412* | *279* |
| Activities of daily living | N | 4,306 | 4.661 |
|  | Difficulty with 1+ | 959 (22.3) | 1,228 (26.4) |
|  | *Missing(N)* | *357* | *2* |
| Limiting long-standing illness | N | 4303 | 4661 |
|  | No | 1982 (46.1) | 1848(39.7) |
|  | Yes, not limiting | 996 (23.2) | 947 (20.3) |
|  | Yes, limiting | 1325 (30.8) | 1866 (40.0) |
|  | *Missing(N)* | *360* | *2* |
| **GEOGRAPHICAL CHARACTERISTICS** | | | |
| Index of Multiple Deprivation | N | 4302 | 4478 |
|  | 1^st^ quintile | 1170 (27.2) | 1163 (26.0) |
|  | 2^nd^ | 1074 (25.0) | 1181 (26.4) |
|  | 3^rd^ | 870 (20.2) | 899 (20.1) |
|  | 4^th^ | 726 (16.9) | 735 (16.4) |
|  | 5th | 462 (10.7) | 500 (11.2) |
|  | *Missing(N)* | *361* | *185* |
| Urban/rural distribution |  | 4306 | 4507 |
|  | Urban | 3128 (72.6) | 3271 (72.6) |
|  | Town/fringe | 546 (12.7) | 565 (12.5) |
|  | Village | 469 (10.9) | 497 (11.0) |
|  | Hamlets /isolated dwellings | 163 (3.8) | 174 (3.9) |
|  | *Missing(N)* | *357* | *156* |
| Geographical regions | N | 4302 | 4496 |
|  | London | 376 (8.7) | 385 (8.6) |
|  | North East | 275 (6.4) | 276 (6.1) |
|  | North West | 479 (11.1) | 503 (11.2) |
|  | Yorkshire and The Humber | 480 (11.2) | 487 (10.8) |
|  | East Midlands | 462 (10.7) | 478 (10.6) |
|  | West Midlands | 469 (10.9) | 494 (11.0) |
|  | East of England | 553 (12.9) | 580 (12.9) |
|  | South East | 715 (16.6) | 761 (16.9) |
|  | South West | 493 (11.5) | 532 (11.8) |
|  | *Missing(N)* | *361* | *167* |
| **LONELINESS REPORT** | | | |
| Individual-based loneliness | N | 3857 | 3891 |
|  | No | 3119 (80.9) | 3208 (82.5) |
|  | Yes (6+ out of 9) | 738 (19.1) | 683 (17.6) |
|  | *Missing(N)* | *806* | *772* |
| Area-based loneliness | N | 3845 | 3890 |
|  | No | 2891 (75.2) | 2993 (75.4) |
|  | Yes (4+ out of 7) | 954 (24.8) | 957 (24.6) |
|  | *Missing(N)* | *818* | *773* |

Individual-based loneliness: UCLA scale =’University California Los Angeles’ loneliness scale (3-question version)

Area-based loneliness: statement to ‘Feel lonely living in this area’ on 7-point Likert scale

**Supplementary file, table 2: Comparison of wave 7 of analytical sample (those participating in waves 3, 6, and 7) and those who dropped-out**

|  |  | Participation in waves 3, 6, and 7 | |
| --- | --- | --- | --- |
|  |  | Yes | No |
|  |  | % /mean (S.D) | |
| Gender | Males | 43.8 | 45.1 |
| Age | In Wave 7 | 72.8 (7.1) | 61.4 (8.5) |
| SOCIAL NETWORK |  |  |  |
| Marital status | (Re)married/partnership | 62.3 | 70.5 |
|  | Lives alone | 15.4 | 22.2 |
|  | Widowed/partner died | 22.4 | 7.2 |
| Has close relationship with 2+ family member/friend | Yes | 87.5 | 92.0 |
|  | No | 12.5 | 8.0 |
| Active civic participation | Yes | 74.2 | 67.4 |
|  | No | 25,8 | 32.6 |
| Part of job market | Out of job market | 84.4 | 43.4 |
|  | Employed/semi-employed | 11.4 | 50.8 |
|  | Looking after home/family | 4.2 | 5.8 |
| HEALTH STATUS |  |  |  |
| Self-rated health | Excellent /very good/good | 70.3 | 77.4 |
|  | Fair/poor | 29.7 | 22.6 |
| Depressive symptoms | No | 80.4 | 80.7 |
|  | 3+ out of 8 | 19.6 | 19.3 |
| Activities of daily living (ADL/IADL) | No difficulties | 73.6 | 82.3 |
|  | Difficulty with 1+ | 26.4 | 17.7 |
| Long-standing illness | No illness | 39.7 | 50.9 |
|  | Yes/Yes and limiting | 60.3 | 49.1 |
| Reported loneliness  (UCLA scale) | No | 82.5 | 82.5 |
|  | Yes (6+ out of 9) | 17.6 | 17.5 |
| Feel lonely living in this area | No | 75.4 | 75.0 |
|  | Yes (4+ out of 7) | 24.6 | 25.0 |

**Supplementary file, table 3. Bivariate regression analyses between two measures of loneliness and socio-demographic characteristics**

|  | | UCLA | Feel lonely living in this area |
| --- | --- | --- | --- |
|  |  | OR (95%CI),  p-value | OR (95%CI),  p-value |
| Gender (ref=Male) | Female | 1.62 (1.4-1.9), <0.001 | 1.7 (1.5-2.0), <0.001 |
| Age | Per 1 year + | 1.02 (1.01-1.03), <0.001 | 1.02 (1.01-1.03), 0.001 |
| **SOCIAL NETWORK** |  |  |  |
| Marital status  (ref= (Re)married/in partnership) | Always single | 2.5 (1.7-3.6), <0.001 | 1.4 (0.9-1.9), 0.096 |
|  | Divorced/separated | 3.2 (2.5-4.2), <0.001 | 2.1 (1.7-2.6), <0.001 |
|  | Widowed/partner died | 3.7 (3.1-4.6), <0.001 | 2.3 (2.0-2.8), <0.001 |
| Has close relationship with 2+ family members/Friends (ref= No) | Yes | 0.4 (0.3-0.4), <0.001 | 0.6 (0.5-0.7), <0.001 |
| Active civic participation (ref= No) | Yes , in 1+ activities | 0.6 (0.5-0.7), <0.001 | 0.6 (0.5-0.8), <0.001 |
| Part of job market  (ref= Out of job market) | Employed/semi-employed | 0.7 (0.5-0.9), 0.011 | 0.8 (0.6-1.0), 0.024 |
|  | Looking after home/family | 1.1 (0.7-1.6), 0.642 | 1.2 (0.9-1.8), 0.220 |
| **HEALTH STATUS** |  |  |  |
| Self-rated health  (ref= Excellent/very good/good) | Fair/poor | 2.7 (2.3-3.2), <0.001 | 1.9 (1.6-2.2), <0.001 |
| Depressive symptoms (ref= No) | 3+ out of 8 | 7.4 (6.1-8.9), <0.001 | 2.9 (2.4-3.5), <0.001 |
| Activities of daily living ADL/IADL  (ref=No difficulties) | Difficulty with 1+ activity | 2.4 (2.0-2.8), <0.001 | 1.6 (1.4 -1.9), <0.001 |
| Long-standing illness (ref= No) | Yes, not limiting | 1.1 (0.9-1.4), 0.334 | 1.2 (1.0-1.5), 0.039 |
|  | Yes, limiting | 2.0 (1.7-2.4), <0.001 | 1.6 (1.4-1.9), <0.001 |

**Supplementary file, table 4. Bivariate regression analyses between two measures of loneliness and geographical characteristics**

|  | | UCLA | Feel lonely living in this area |
| --- | --- | --- | --- |
|  |  | OR (95%CI),  p-value | OR (95%CI),  p-value |
| Index of Multiple Deprivation (IMD)  (ref= 1^st^ quintile-least deprived) | 2^nd^ quintile | 1.4 (1.1-1.9), 0.006 | 1.3 (1.0-1.6), 0.018 |
|  | 3^rd^ quintile | 1.4 (1.1-1.8), 0.016 | 1.3 (1.1-1.7), 0.013 |
|  | 4^th^ quintile | 1.6 (1.2-2.1), 0.001 | 1.7 (1.3-2.1), <0.001 |
|  | 5^th^ quintile  (most deprived) | 2.0 (1.5-2.7), <0.001 | 1.9 (1.5-2.5), <0.001 |
|  | *P for trend* | *<0.001* | *<0.001* |
| Urban/rural distribution  (ref= Urban) | Town/fringe | 1.0 (0.8-1.3), 0.986 | 0.8 (0.6-1.0), 0.044 |
|  | Village | 0.8 (0.6-1.1), 0.178 | 0.9 (0.7-1.1), 0.320 |
|  | Hamlets /isolated dwellings | 0.9 (0.6-1.4), 0.539 | 0.9 (0.6-1.3), 0.559 |
|  | *P for trend* | *0.206* | *0.137* |
| Geographical regions  (ref= London) | North East | - 1. (0.7-1.9), 0.591 | 0.7 (0.5-1.1), 0.115 |
|  | North West | 1.5 (1.0-2.3), 0.058 | 0.8 (0.6-1.1), 0.193 |
|  | Yorkshire and The Humber | 1.6 (1.1-2.5), 0.023 | 1.0 (0.7-1.4), 0.889 |
|  | East Midlands | 1.6 (1.0-2.4), 0.041 | 0.8 (0.6-1.1), 0.233 |
|  | West Midlands | 1.6 (1.1-2.5), 0.022 | 0.9 (0.6-1.2), 0.442 |
|  | East of England | 1.2 (0.8-1.8), 0.304 | 1.0 (0.7-1.4), 0.850 |
|  | South East | 1.2 (0.8-2.6), 0.311 | 1.0 (0.7-1.3), 0.875 |
|  | South West | 1.7 (1.1-2.6), 0.013 | 1.0 (0.7-1.4), 0.873 |

**Supplementary file, table 5: Loneliness by geographical characteristics in waves 3 and 7**

|  | | Wave 3 | | Wave 7 | |
| --- | --- | --- | --- | --- | --- |
|  |  | Individually-based loneliness (%) | Area-based loneliness (%) | Individually-based loneliness (%) | Area-based loneliness (%) |
| Index of Multiple Deprivation | 1^st^ quintile (least deprived) | 15.1 | 21.9 | 13.3 | 19.4 |
|  | 2^nd^ | 18.1 | 23.7 | 17.7 | 23.8 |
|  | 3^rd^ | 19.2 | 24.1 | 17.4 | 24.3 |
|  | 4^th^ | 21.8 | 29.5 | 19.5 | 28.9 |
|  | 5th | 28.8 | 29.9 | 23.2 | 31.6 |
| Urban/rural distribution | Urban | 19.5 | 25.7 | 17.7 | 25.3 |
|  | Town/fringe | 19.0 | 23.4 | 17.7 | 21.0 |
|  | Village | 18.2 | 23.0 | 15.1 | 23.1 |
|  | Hamlets /isolated dwellings | 15.1 | 17.8 | 15.8 | 23.3 |
| Geographical regions | London | 17.5 | 25.5 | 13.0 | 25.9 |
|  | North East | 16.9 | 21.0 | 14.6 | 20.1 |
|  | North West | 20.8 | 21.6 | 18.3 | 21.8 |
|  | Yorkshire and The Humber | 20.5 | 26.5 | 19.5 | 26.4 |
|  | East Midlands | 22.1 | 26.0 | 18.8 | 22.0 |
|  | West Midlands | 19.8 | 24.0 | 19.6 | 23.4 |
|  | East of England | 16.2 | 25.8 | 15.6 | 26.5 |
|  | South East | 18.2 | 25.0 | 15.5 | 25.5 |
|  | South West | 20.0 | 26.3 | 20.1 | 25.4 |

Individual- based loneliness=UCLA scale =’University California Los Angeles’ loneliness scale (3-question version)

Area-based loneliness= ‘How much do you feel lonely living in this area?”

**Supplementary file, table 6 a,b: Cross-tabulation of reported loneliness measures (R-UCLA and ‘Often feel lonely living in this area’) by wave (N (%))**

1. % of the whole sample

|  | | Area-based loneliness | | | | | |
| --- | --- | --- | --- | --- | --- | --- | --- |
|  |  | **Wave 3** | | | **Wave 7** | | |
|  |  | No | Yes | Total | No | Yes | Total |
| Individual-based loneliness | No | 2,559 (67.0) | 538 (14.1) | 3,097 (81.0) | 2,561 (67.7) | 566 (15.0) | 3,127 (82.6) |
|  | Yes | 317 (8.3) | 408 (10.7) | 725 (19.0) | 303 (8.0) | 355 (9.4) | 658 (17.4) |
|  | Total | 2,876 (75.3) | 946 (24.8) | 3,822 (100.0) | 2,864 (75.7 ) | 921 (24.3 ) | 3,785 (100.0) |

b) % of those in particular area-based loneliness category

|  | | Area-based loneliness | | | | | |
| --- | --- | --- | --- | --- | --- | --- | --- |
|  |  | **Wave 3** | | | **Wave 7** | | |
|  |  | No | Yes | Total | No | Yes | Total |
| Individual-based loneliness | No | 2,559 (89.0) | 538 (56.9) | 3,097 (81.0) | 2,561 (89.4) | 566 (61.5) | 3,127 (82.6) |
|  | Yes | 317 (11.0) | 408 (43.1) | 725 (19.0) | 303 (10.6) | 355 (38.6) | 658 (17.4) |
|  | Total | 2,876 (100.0) | 946 (100.0) | 3,822 (100.0) | 2,864 (100.0) | 921 (100.0) | 3,785 (100.0) |

**Supplementary file, table 7: Cross-tabulation of urban/rural characteristic and IMD (in %), wave 6**

| Urban/rural characteristic | Index of Multiple Deprivation | | | | | TOTAL |
| --- | --- | --- | --- | --- | --- | --- |
|  | Least deprived | 2^nd^ quintile | 3^rd^ quintile | 4^th^ quintile | Most deprived |  |
| Urban | 17.8 | 15.7 | 14.4 | 14.1 | 10.7 | 72.7 |
| Town & Fringe | 4.6 | 4.8 | 1.7 | 1.1 | 0.4 | 12.5 |
| Village | 2.5 | 4.8 | 2.8 | 0.9 | 0.1 | 11.0 |
| Hamlet & Isolated dwelling | 1.1 | 1.1 | 1.3 | 0.3 | 0 | 3.7 |
| TOTAL | 26.0 | 26.4 | 20.1 | 16.4 | 11.2 | 100% |
